# A Sensitive FRET Biosensor Based on Carbon Dots-Modified Nanoporous Membrane for 8-hydroxy-2′-Deoxyguanosine (8-OHdG) Detection with Au@ZIF-8 Nanoparticles as Signal Quenchers

**DOI:** 10.3390/nano10102044

**Published:** 2020-10-16

**Authors:** Weiwei Ye, Yu Zhang, Wei Hu, Liwen Wang, Yu Zhang, Ping Wang

**Affiliations:** 1 Key Laboratory of E&M, MOE, Zhejiang University of Technology, Hangzhou 310023, China; 2 College of Mechanical Engineering, Zhejiang University of Technology, Hangzhou 310023, China; 3 Department of Food Science and Technology, Zhejiang University of Technology, Hangzhou 310014, China; zhangyuzjut@163.com (Y.Z.); weihu1995@163.com (W.H.); Wlw4712@163.com (L.W.); 4 Mechanical and Automotive Engineering, School of Engineering, RMIT University, Melbourne, Victoria 3004, Australia; fiona.zhang@rmit.edu.au; 5 Biosensor National Special Laboratory, Key Laboratory for Biomedical Engineering of Education Ministry, Department of Biomedical Engineering, Zhejiang University, Hangzhou 310027, China; cnpwang@zju.edu.cn

**Keywords:** nanoporous alumina membrane, fluorescence resonance energy transfer, Au@ZIF-8 nanoparticles, DNA oxidation damage, 8-OHdG detection

## Abstract

A sensitive fluorescence resonance energy transfer (FRET) biosensor is proposed to detect 8-hydroxy-2′-deoxyguanosine (8-OHdG), which is a typical DNA oxidation damage product excreted in human urine. The FRET biosensor was based on carbon dots (CDs)-modified nanoporous alumina membrane with CDs as fluorescence donors. Gold nanoparticles were encapsulated in zeolitic imidazolate framework-8 to form Au@ZIF-8 nanoparticles as signal quenchers. CDs and Au@ZIF-8 nanoparticles were biofunctionalized by 8-OHdG antibody. The capture of 8-OHdG on the membrane substrates can bring Au@ZIF-8 nanoparticles closely to CDs. With 350 nm excitation, the fluorescence of CDs was quenched by Au@ZIF-8 nanoparticles and FRET effect occurred. The quenching efficiency was analyzed. The limit of detection (LOD) was 0.31 nM. Interference experiments of the FRET biosensor showed good specificity for 8-OHdG detection. The biosensor could detect urinary 8-OHdG sensitively and selectively with simple sample pretreatment processes. It shows applicability for detecting biomarkers of DNA damage in urine or other biological fluids.

## 1. Introduction

The potential toxicity of environmental pollutants and pharmaceutical candidates to human health relates to their metabolites reactive oxygen species (ROS) [1,2]. They cause the damage of genetic materials, which refers to genotoxicity, giving rise to carcinogenesis, diabetes, aging, neurodegenerative, and cardiovascular diseases. Guanine has the lowest oxidation potential among the four nucleobases of the DNA molecule, and it is most susceptible to oxidative damage [3]. In particular, 8-hydroxy-2′-deoxyguanosine (8-OHdG) is an oxidized product of the deoxyguanosine residues in DNA, which is formed from the attack of hydroxyl radicals (•OH) at the C-8 position of guanine [4]. Thus, 8-OHdG molecules are excreted into urine. The urinary 8-OHdG reflects the extent of oxidative DNA damage in human body [5]. Hence, it has an impact on analytical science to develop a sensitive and selective platform for 8-OHdG determination and evaluation.

A variety of analytical methods, such as resonance Rayleigh scattering (RRS), high-performance liquid chromatography with electrochemical detection (HPLC-ED), gas chromatography-mass spectrometry (GC-MS), capillary electrophoresis (CE), and enzyme-linked immunosorbent assay (ELISA), have been developed for 8-OHdG investigation [6,7,8,9]. These methods can determine 8-OHdG with good selectivity and suitable detection limit, but most of them suffer from expensive instrument, high requirement of sample pretreatment, and operation processes. Some biosensor devices based on electrochemical or electrochemiluminescence detection modes have been developed for 8-OHdG detection [10]. Importantly, 8-OHdG can be directly read making use of active materials or highly conductive materials of carbon nanotubes or graphene modified on electrodes. Electrochemical sensors have obvious advantages including low cost, easy miniaturization, and simplicity, but their performance is affected by material properties of electrodes [11]. Fluorescence resonance energy transfer (FRET) technology has been served as the designing strategy of various biosensors for medical diagnosis, food safety, and environmental detection due to its high sensitivity, facile operation, and high specificity [12]. A luminescent paper-based device and a fluorescence aptasensor were developed for oxidative stress biomarker 8-OHdG detection [13,14]. The fluorescence signals relied on chemical fluorophore, which had the disadvantage of photobleaching. 

Nanoscale metal−organic frameworks (nMOFs) emerge as an important class of nanomaterials in diverse fields because of their charming properties of high porosity, structural diversity, and multifunctionality. They have attracted much attention in areas of gas storage and separation, energy conversion, and sensing [15,16,17]. However, it should be noted that the research of nMOFs in the sensing field should still be further explored deeply in the future. Zeolitic imidazolate framework-8 (ZIF-8) is a kind of the typical nMOFs materials [18,19]. It is composed of ZnN4 tetrahedral structure unit formed by zinc ion (Zn^2+^) and nitrogen atom (N) in methylimidazole ester. It has a zeolite structure with large pores and shows simple crystal structure, good stability, and high loading capacity. ZIF-8 nanocrystals are used as the host material. Both gold nanoparticles and silver nanoparticles can be used as guests to be encapsulated in ZIF-8 nanocrystals to form Au@ZIF-8 nanoparticles and Ag@ZIF-8 nanoparticles, respectively [20]. These structures avoid nanoparticles being disturbed by the environment. Compared to Ag@ZIF-8 nanoparticles, the synthesis process of Au@ZIF-8 nanoparticles can be easily controlled and the size of gold nanoparticles dispersed on ZIF-8 crystals is uniform. Au@ZIF-8 nanoparticles have property synergies of inorganic nanoparticles and MOFs for multifunctional applications. They combine the advantages of structural adaptivity, flexibility, high specific surface area, and high fluorescence quenching efficiency [21,22]. Au@ZIF-8 nanoparticles are candidates for highly sensitive molecule detection. Unsaturated metal site makes it easy to be modified for biosensing, targeted imaging, and therapy.

Since biosensor detection is mainly performed on solid substrate. Nanoporous alumina membranes are covered with through nanopores, which make the membranes have high surface-to-volume ratio property. Together with the advantages of easy fabrication and surface biofunctionalization, nanoporous membranes are good substrates for constructing biosensors to detect neurotoxin, nucleic acid, proteins, virus, and food hazards [23,24,25,26,27,28]. Nanoporous alumina membrane-based FRET biosensors with nanoparticles as the fluorescence donors and acceptors provide easy operation, good photostability, and sensitivity for 8-OHdG detection. Traditional semiconducting quantum dots are toxic and cause environmental pollution due to the heavy metal components. Organic dyes as fluorescent probes can be easily obtained but suffer from photobleaching [29]. Carbon dots (CDs) emerge as superior fluorophores due to their advantageous virtues, such as low cost, excellent photostability, nontoxicity, and ease to be modified with biomolecules [30,31]. These advantageous merits make CDs attractive candidates in the areas of sensing and catalysis applications [32,33,34,35,36,37]. CDs together with gold nanoparticles have been used to develop a FRET assay method for detecting DNA containing oxidatively damaged product 8-OHdG (DNA-8-OHdG) [38]. CDs and gold nanoparticles acted as the donor and acceptor pair. The detection process was performed in the solution. CDs modified on nanoporous alumina membranes can form good substrates with fluorescence and extends the assay to the solid substrate for 8-OHdG biosensing. 

In the study, we develop a sensitive FRET biosensor based on CDs-modified nanoporous alumina membrane with CDs as fluorescence donors and Au@ZIF-8 nanoparticles as signal quenchers for 8-OHdG detection in urine. Nanoporous alumina membranes were conjugated with CDs, which were modified with glutaraldehyde for 8-OHdG antibody conjugation. CDs-modified nanoporous alumina membranes were used as the biosensor substrate for 8-OHdG detection. Au@ZIF-8 nanoparticles were functionalized with 8-OHdG antibody. CDs and Au@ZIF-8 nanoparticles acted as the fluorescence donors and signal quenchers, respectively. The addition of 8-OHdG to the biosensor substrate can bring Au@ZIF-8 nanoparticles closely to CDs. The emission of CDs under 350 nm photoexcitation was quenched by Au@ZIF-8 nanoparticles leading to FRET effect on the nanoporous alumina membrane substrate. Further, 8-OHdG can be detected by calculating the fluorescence intensity change with FRET effect. The limit of detection (LOD) of this FRET biosensor for 8-OHdG detection is 0.31 nM. It shows the potential applications for sensitive DNA damage biomarker detection.

## 2. Materials and Methods 

### 2.1. Materials

Specifically, 8-OHdG, bovine serum albumin (BSA), citric acid, diethylenetriamine (EDTA), (3-glycidyloxypropyl)trimethoxysilane (GPMS), glutaraldehyde, methylbenzene, chloroauric acid, 3-mercaptopropionic acid (MPA), zinc nitrate hexahydrate (Zn(NO_3_)_2_·6H_2_O), methanol (99.8%), sodium chloride, potassium chloride, calcium chloride, acetone, N,N-dimethylformamide (DMF), sodium borohydride (NaBH_4_, 99.99%), and dehydrated alcohol were obtained from Sigma Aldrich (St. Louis, Missouri (Mo), USA). NaCl, KCl, CaCl_2_, MgCl_2_, thymine, cytosine, adenine, guanine, and hydrogen peroxide were ordered from ALADDIN Reagent (Shanghai, China). Nanoporous alumina membranes were purchased from Whatman (Boston, Massachusetts (Ma), USA). Alongside, 8-OHdG antibody was bought from Abcam (Cambridge, UK). In addition, 2-methylimidazole (2-MeIM, 99%), 1-dodecanethiol (DDT, ≥98%), hexadecyltrimethyl ammonium bromide (CTAB, 98%), silver nitrate (AgNO_3_, ≥99.0%), 11-mercaptoundecanoic acid (MUA, 95%), and 4-nitrophenol (4-NP, ≥99.5%) were purchased from Sigma Aldrich (St. Louis, Missouri (Mo), USA). The chemicals were used as received without further purification.

### 2.2. Synthesis of Carbon Dots (CDs) 

CDs were synthesized with citric acid and EDTA by one-step hydrothermal treatment [38]. Citric acid (0.22 g) was dissolved in deionized water (DI water, 10 mL). EDTA (112.6 μL) was added to citric acid solution. The mixture was stirred thoroughly, transferred, and sealed into a clean and dry Teflon equipped stainless steel autoclave. Hydrothermal treatment at 200 °C for 6 h was carried out on it and then was cooled down naturally to room temperature. After centrifugation of dark brown solution at 13.200 rpm/min for 15 min, the supernatant was freeze-dried to collect CDs. CDs (1 mg) were dissolved in DI water (1 mL) and stored for later use. 

### 2.3. Surface Functionalization of Nanoporous Alumina Membrane

Nanoporous alumina membranes, which had pore diameters of 200 nm, were surface functionalized according to the procedures in our previous studies [23]. They were boiled in hydrogen peroxide for 30 min to form hydroxyl groups on the surface and rinsed by DI water. After gentle shaking for 15 min, nanoporous membranes were dried and immersed in solution of GPMS and methylbenzene (2%). They were sealed and reaction was carried out at 60 °C for 24 h. They were washed 3 times by acetone and absolute ethyl alcohol. The membranes were cured at 60 °C for 2 h and stored with desiccation. CDs solution (0.1 mg/mL, 150 μL) was added to the as-prepared silanized nanoporous alumina membranes, and kept overnight in moist and dark environment at room temperature. The membranes were rinsed in DI water. Glutaraldehyde (10 μL) was added to membranes for 30 min to activate amino group of CDs that were immobilized on membranes. After that, 8-OHdG antibody solution (1 μg/mL, 150 μL) was added to membranes and incubated overnight at 4 °C. Further, 8-OHdG antibody was conjugated on CDs with glutaraldehyde as linker. The functionalized nanoporous membranes were cleaned by three repeated wash cycles and stored at 4 °C.

### 2.4. Synthesis of Au@ZIF-8 Nanoparticles

Au@ZIF-8 nanoparticles were prepared according to previous study with slight modification [20]. Chloroauric acid and MPA were dissolved in methanol solution with the concentrations of 0.01 and 0.1 M, respectively. Chloroauric acid solution (1.5 mL) and MPA solution (0.45 mL) were mixed, stirring for 3 h at room temperature. The formed Au-MPA compounds was collected and purified by centrifugation (10,000 rpm/min, 10 min) followed by washing with methanol twice. The precipitate was resuspended in methanol solution (4 mL). Zinc nitrate and 2-methyl imidazole were dissolved in DMF with the concentrations of 0.070 and 0.43 M, respectively. The zinc nitrate solution (4.8 mL) was added to the same volume of 2-methyl imidazole solution, stirring for 5 min. The mixture was pipetted and transferred to a dry Teflon equipped stainless steel autoclave (100 mL). The reaction lasted for 6 h at 120 °C. ZIF-8 was synthesized and collected by centrifugation (10,000 rpm/min, 10 min). It was washed by methanol twice and resuspended in 10 mL methanol solution. The as-prepared Au-MPA solution (2 mL) was diluted with methanol solution. The freshly prepared methanol solution of R-NBH_4_ (4 mL, 0.25 M) was added to Au-MPA solution, stirring for 15 min. Then, the as-prepared ZIF-8 solution (8 mL) was mixed with the above solution and stirred for 1 h to form Au@ZIF-8 nanoparticles. They were collected by centrifugation (10,000 rpm/min, 10 min) and washed twice by methanol. The precipitate was resuspended in 16 mL methanol solution for later use. 

### 2.5. Surface Biofunctionalization of Au@ZIF-8 Nanoparticles

Au@ZIF-8 nanoparticle solution was centrifuged with the speed of 8000 rpm/min for 10 min and rinsed twice by DI water. Au@ZIF-8 nanoparticles were resuspended in DI water. After the solution was repeatedly diluted 4 times, Au@ZIF-8 nanoparticle solution was mixed with 8-OHdG antibody (5 μL/mL) and incubated at 4 °C overnight. The mixture was purified by centrifugation (8000 rpm/min, 10 min) followed by DI water rinsing procedure. The biofunctionalized Au@ZIF-8 nanoparticles were blocked by BSA (1%). The mixture was centrifuged (8000 rpm/min, 10 min) and rinsed 3 times by DI water. The precipitate was dispersed in phosphate buffer saline (PBS) for later use.

### 2.6. Characterization

Maximum excitation wavelength of synthetic CDs was determined by F2700 (Hitachi, Japan). Transmission electron microscopy (TEM) was applied to investigate the morphologies and sizes of Au@ZIF-8 nanoparticles and CDs by JEOL-2100F (JEOL, Japan). Powder X-ray diffraction was used to identify crystallography information by the X’Pert PRO X-ray diffractometer (PANalytical Ltd., Almelo, The Netherlands). Fourier-transform infrared (FTIR) spectra recorded chemical bonding information using Nicolet 6700 spectrometer (Thermo-Fisher, Waltham, MA, USA). The morphology of nanoporous alumina membranes were observed by SU8010 field emission scanning electron microscope equipped with an energy dispersive X-ray spectrometer attachment (SEM, Hitachi, Japan). 

### 2.7. FRET Biosensor for 8-OHdG Detection

To investigate the detection of 8-OHdG by the FRET biosensor, various concentrations of 8-OHdG diluted in PBS (pH 7.4, 40 μL) were added to the functionalized nanoporous alumina membranes. They were reacted for 4 h at 37 °C. The membranes were rinsed to remove the unreacted chemicals. Au@ZIF-8 nanoparticles were modified by 8-OHdG antibody. The detection of 8-OHdG brought Au@ZIF-8 nanoparticles to CDs on nanoporous alumina membranes. The fluorescence intensity for various concentrations of 8-OHdG detection was recorded using F2700 ranging from 400 to 600 nm under 350 nm photoexcitation. The selectivity of the FRET biosensor was studied with the same concentration (3 μM) of NaCl, KCl, CaCl_2_, MgCl_2_, thymine, cytosine, guanine, and adenine as interferences.

### 2.8. Detection of 8-OHdG in Urine Samples

Importantly, 8-OHdG was one kind of metabolites that appeared in urine. Urine samples were obtained from healthy young volunteers with consent. The protocol was approved by the ethics committee of Sir Run Run Shaw Hospital, an affiliate of Medical College, Zhejiang University (approval number: 20200831-34). Urine samples were collected and processed within an hour. They were centrifuged with the speed of 5000 rpm/min for 10 min. The supernatant was filtered by a 0.22 μm membrane diluted by PBS (pH = 7.4) with the dilution ratio of two times. The diluted supernatant (100 μL) was added to the FRET biosensor for 8-OHdG detection. 

## 3. Results

### 3.1. Principle of FRET Biosensor

**Figure 1:**
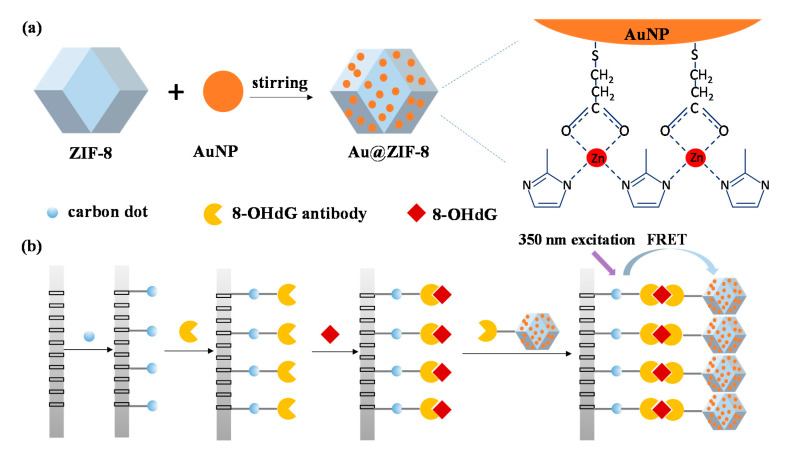
Schematic representation of the synthesis process of Au@ZIF-8 nanoparticles (**a**) and 8-hydroxy-2′-deoxyguanosine (8-OHdG) detection by nanoporous alumina membranes-based fluorescence resonance energy transfer (FRET) biosensor with carbon dots (CDs) and Au@ZIF-8, respectively, as fluorescence donors and acceptors (**b**).

Figure 1 shows the principle of 8-OHdG detection by CDs-modified nanoporous alumina membrane-based FRET biosensor with CDs and Au@ZIF-8 nanoparticles as fluorescence donors and signal quenchers, respectively. The synthesis process of Au@ZIF-8 nanoparticles is shown in Figure 1a. Au@ZIF-8 nanoparticles were synthesized with incorporation of gold nanoparticles into ZIF-8 structures by stirring. Au@ZIF-8 nanoparticles combined the advantages of both ZIF-8 structures and gold nanoparticles. They had microporous properties and wide absorption spectra. Nanoporous alumina membranes were functionalized and immobilized with CDs. CDs were modified with glutaraldehyde for 8-OHdG antibody conjugation. Thus, 8-OHdG molecules were specifically captured by antibody on CDs and anchored onto nanoporous alumina membranes. Au@ZIF-8 nanoparticles were biofunctionalized by 8-OHdG antibody and brought Au@ZIF-8 nanoparticles closely to CDs. With 350 nm photoexcitation, the fluorescence energy of CDs transferred to Au@ZIF-8 nanoparticles causing FRET effect (Figure 1b). By recording and calculating the fluorescence intensity change, various concentrations of 8-OHdG can be detected. 

### 3.2. Characterization of CDs and Au@ZIF-8 Nanoparticles

**Figure 2:**
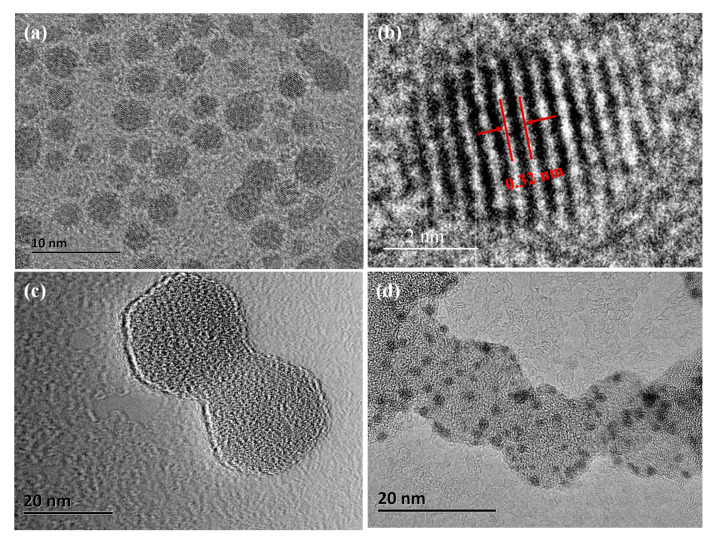
TEM images of (**a**) spherical CDs, (**b**) high-resolution TEM (HRTEM) image of the CDs, (**c**) ZIF-8 nanocrystals, and (**d**) Au@ZIF-8 nanoparticles.

The sizes and morphology of Au@ZIF-8 nanoparticles and CDs were characterized by TEM. The CDs have spherical shape and disperse well in DI water (Figure 2a). The average diameter of CDs is about 5 nm. They have the d spacing values of 0.32 nm shown from the high-resolution TEM (HRTEM) image (Figure 2b). It is near the (002) planes of graphitic carbon [39]. ZIF-8 nanocrystals are polyhedron and the average diameter is about 20 nm (Figure 2c). The ZIF-8 shell thickness was affected by the initial solution concentration or the surfactants [20]. Gold nanoparticles were capped with biofunctional MPA. The thiol groups of MPA had strong interaction with gold nanoparticles. The integration of gold nanoparticles to ZIF-8 nanocrystals was based on the coordination interaction between the carboxylate anions of MPA and the unsaturated Zn^2+^ cations on the exterior surface of ZIF-8. MPA was an important linker to stabilize gold nanoparticles onto the surfaces of ZIF-8 nanocrystals. A thin layer of gold nanoparticles with the diameter of 3 nm are uniformly distributed on the ZIF-8 nanocrystal surfaces forming Au@ZIF-8 nanoparticles (Figure 2d). 

Analysis by XRD was done to characterize the phase composition of Au@ZIF-8 nanoparticles. Figure S1 shows the XRD pattern of Au@ZIF-8 nanoparticles. The XRD patterns of Au@ZIF-8 nanoparticles have diffraction peaks at 7.3°, 10.4°, 12.7°, 14.7°, 16.4°, and 18.0°, which are matched with the crystallographic plane of (011), (002), (112), (022), (013), and (222), respectively [40]. The XRD pattern of ZIF-8 nanocrystals was strong and the ultrafine size of AuNPs was difficult to be observed by the XRD technique. 

FTIR spectra of Au@ZIF-8 nanoparticles are shown in Figure S2. The wide peak at 3429.4 cm^−1^ indicates -OH, which may be due to the residual moisture in the material. The peak at 2925 cm^−1^ is associated with the stretching vibration of aromatic C-H in imidazole. The peak at 1574 cm^−1^ represents the stretching vibration of C=N. The characteristic peak at 1458 cm^−1^ indicates the methyl bending vibration. The peaks at 1145 and 997 cm^−1^ can be attributed to the stretching vibration of C-N in imidazole ring. The Zn–N stretching of ZIF-8 crystal is shown at 420 cm^−1^. The FTIR spectra confirm the main chemical bond of Au@ZIF-8 nanoparticles [41].

### 3.3. Morphologies of Sensing Surfaces

**Figure 3:**
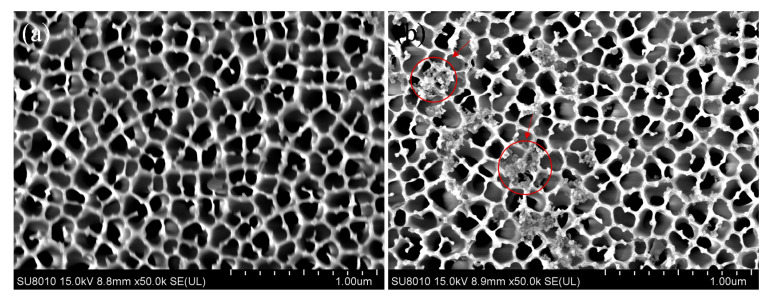
SEM images of nanoporous alumina membranes without 8-OHdG captured (**a**) and with 8-OHdG captured on nanoporous alumina membranes (**b**).

The surface morphologies of nanoporous alumina membranes without 8-OHdG captured and with 8-OHdG captured on nanoporous alumina membranes are shown in Figure 3. A thin platinum layer was sputtered on sample surfaces by focused ion beam (FIB) induced deposition to increase the conductivity for SEM observation. Honeycomb-like nanoporous membranes become rough after silanization and modification with CDs (Figure 3a). CDs were conjugated on nanoporous alumina membranes by covalent bonding. A single CD was too small to be observed under SEM. Moreover, 8-OHdG molecules were captured by 8-OHdG antibody that was modified on CDs. Au@ZIF-8 nanoparticles were used to label 8-OHdG molecules on nanoporous alumina membranes. Figure 3b shows that the surfaces of nanoporous alumina membranes are covered by small agglomerated particles, which are indicated by red arrows. It is due to the conjugation of Au@ZIF-8 nanoparticles. Energy-dispersive X-ray spectroscopy (EDX) result for the nanoporous alumina membranes with 8-OHdG capture and Au@ZIF-8 nanoparticles conjugation is shown in Figure S3. Prominent signals of Zn and Au are shown in the spectrum, showing the successful capture of 8-OHdG and labeling of Au@ZIF-8 nanoparticles.

### 3.4. Optimization of the Experimental Conditions

CDs have wide emission spectra ranging from violet to yellow and the emission intensity is pH dependent [24]. The emission spectrum of present CDs is between 400 and 550 nm with the excitation wavelength between 300 and 400 nm (Figure S4). The best excitation wavelength and emission wavelength of CDs were found as 350 and 440 nm, respectively. The CDs suspension shows strong blue light emission with the excitation of 350 nm wavelength (Figure S4 inset). The photoluminescence of CDs varies with different pH conditions (Figure S5). The CDs suspension emits blue light (Figure S5 inset) and the fluorescence intensity is low in relatively low pH solution. The fluorescence intensity is also low in alkaline solution in comparison with CDs in neutral buffer. The pH-sensitive properties of CDs depend on ionization of surface functional groups [42]. The most suitable working condition of CDs is in neutral buffer with pH 7.4.

### 3.5. Detection of 8-OHdG by the FRET Biosensor

**Figure 4:**
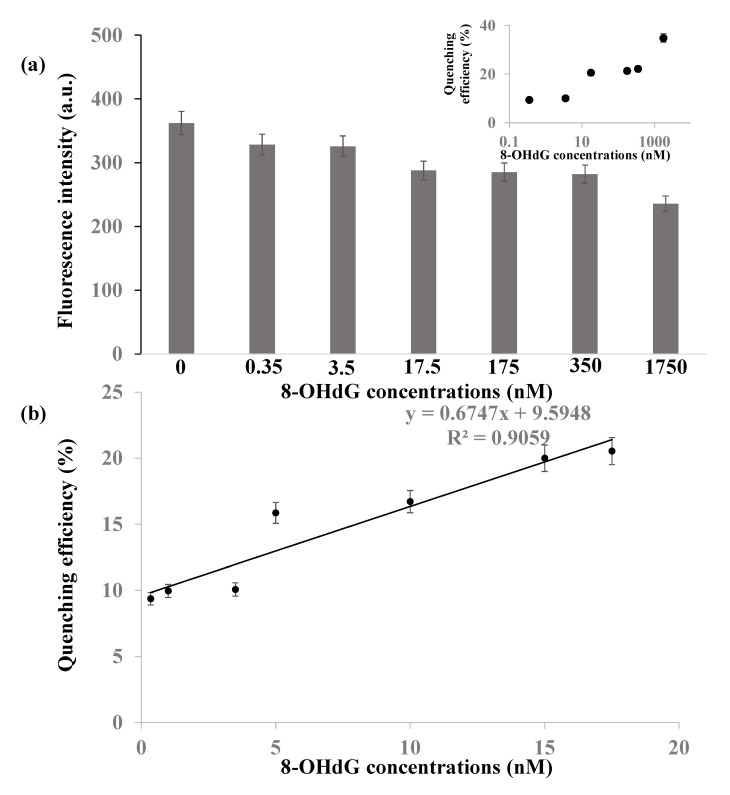
(**a**) Fluorescence intensity change for different concentrations of 8-OHdG detection and the quenching efficiency of 8-OHdG detection by the nanoporous alumina membrane-based FRET biosensor (inset). (**b**) The quenching efficiency of 8-OHdG detection with low concentrations.

FRET biosensor was constructed based on nanoporous alumina membrane with CDs and Au@ZIF-8 nanoparticles, respectively, as fluorescence donors and acceptors. To investigate the performance of the biosensor, various concentrations of 8-OHdG were detected. A fixed number of CDs (0.1 mg/mL, 150 μL) was anchored on functionalized nanoporous alumina membranes and biofunctionalized by 8-OHdG antibody. Various concentrations of 8-OHdG molecules can be specifically captured by CDs on nanoporous membranes and labeled by Au@ZIF-8 nanoparticles (0.2 mg/mL, 100 μL) that were modified by 8-OHdG antibody. Figure 4a shows fluorescence intensity change of nanoporous alumina membranes before and after 8-OHdG detection. Fluorescence intensity declined obviously with Au@ZIF-8 nanoparticles addition, showing the FRET phenomenon occurred between CDs and Au@ZIF-8 nanoparticles. As the increase of 8-OHdG concentrations in the range of 0.35–1750 nM, fluorescence intensity decreased more. Figure 4a (inset) shows quenching efficiency for different concentrations of 8-OHdG detection. The quenching efficiency (QE) was calculated by the equation QE% = (F_0_−F_q_)/F_0_ × 100%. It was increased with the increase of 8-OHdG concentrations. Experiments with low concentrations of 8-OHdG were carried on to study the limit of detection (LOD) of this FRET biosensor. Figure 4b shows the fluorescence quenching efficiency versus 8-OHdG concentrations. The relationship between fluorescence quenching efficiency and 8-OHdG concentration was y = 0.6747x + 9.5948 (R^2^ = 0.9059), where y was the fluorescence quenching efficiency and x was the 8-OHdG concentrations (nM). The quenching efficiency had linear relationship with 8-OHdG concentrations in the range of 1–17.5 nM. Three replicated experiments were used in the calculation of standard deviation in error bars. The LOD was determined by the control signals plus three times of noise signals (standard derivation). It was calculated to be 0.31 nM for the CDs-modified nanoporous alumina membrane-based FRET biosensor for 8-OHdG detection.

The performance of the CDs-modified nanoporous alumina membrane-based FRET biosensor with Au@ZIF-8 nanoparticles as fluorescence acceptors for 8-OHdG detection is compared with that of some reported studies in Table 1. It shows a comparative study on 8-OHdG detection by various methods and materials applied. In comparison with previous methods including quartz crystal microbalance (QCM), electrochemical sensors, and fluorescence aptasensors for 8-OHdG detection, the study presented here has the advantages of low LOD, wide detection range, and excellent sensitivity. The reaction time for 8-OHdG sensing in the study is longer than some reported studies [38,43]. It allows 8-OHdG to pass through the membrane pores and react with the antibody in the nanopores. In the future, we can add press to accelerate the passing procedure and short reaction time. 

### 3.6. Specificity of FRET Biosensor

**Figure 5:**
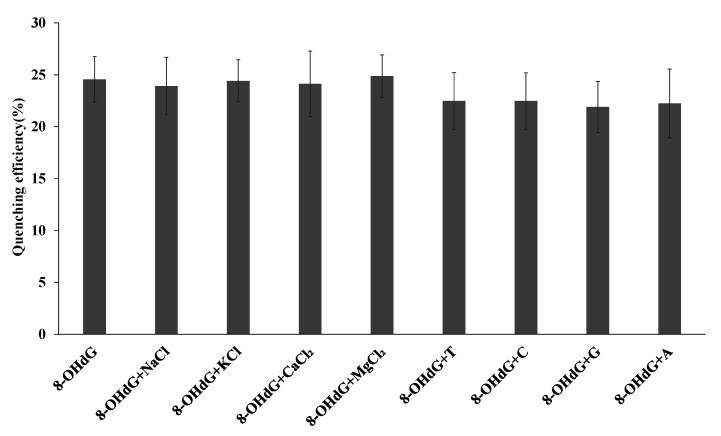
Detection of 8-OHdG in the presence of interference NaCl, KCl, CaCl_2_, MgCl_2_, thymine, cytosine, guanine, and adenine.

Specificity of the FRET biosensor was investigated by parallel addition of different substances such as NaCl, KCl, CaCl_2_, MgCl_2_, thymine, cytosine, guanine, and adenine to the fixed amount of 8-OHdG. Figure 5 indicates the quenching efficiency change of 8-OHdG detection without and with NaCl, KCl, CaCl_2_, MgCl_2_, thymine, cytosine, guanine, and adenine interference. The quenching efficiency of 8-OHdG was 24.6%. With NaCl, KCl, CaCl_2_, MgCl_2_, thymine, cytosine, guanine, and adenine interference, the quenching efficiency became 23.9%, 24.4%, 24.1%, 24.9%, 22.5%, 22.5%, 21.9%, and 22.2%, respectively. The variations were 2.6%, 0.6%, 1.8%, 1.3%, 8.5%, 8.5%, 10.9%, and 9.5% for NaCl, KCl, CaCl_2_, MgCl_2_, thymine, cytosine, guanine, and adenine interference (Figure S6). The error bars were calculated from standard deviation of three replicated experiments. The interference substances had little influence on the specificity of the biosensor, showing good specificity of the nanoporous alumina membrane-based FRET biosensor. The quenching efficiency was 3.8% of control experiment on omitting 8-OHdG from the assay. When functionalized CDs and 8-OHdG were present, but nonfunctionalized Au@ZIF-8 nanoparticles were used, the quenching efficiency was 5.7%. When functionalized Au@ZIF-8 nanoparticles were present along with 8-OHdG, but antibody-free CDs were incorporated into the nanoporous alumina membrane, the quenching efficiency was 2.9%. These control experiments show the good specificity of FRET biosensor towards antibody.

### 3.7. Analysis of Human Urine Samples

In general, 8-OHdG molecules are expressed in body fluids, cells, tissues, and excreted in urine. Urine is easy to collect and 8-OHdG concentration level in urine is stable. Urine samples can be stored for 1 day at 25 °C, for 7 days at 4 °C, and even for 1–2 years at −80 °C [36]. Thus, 8-OHdG is detected in urine to indicate the oxidation of human body. The pH of human urine is in the range of 4.6 and 8.0. The fluorescence intensity of CDs may be decreased obviously in acidic condition. PBS (pH = 7.4) was used to dilute samples to make sure the fluorescence of CDs in different samples. Three groups of diluted urine samples were assayed and recovery experiments were carried out via the standard addition method. The fluorescence intensity was detected for the three diluted samples and the quenching efficiency was calculated. The 8-OHdG concentrations of sample 1, sample 2, and sample 3 were 11.36, 12.02, and 10.54 nM, respectively, showing that the urinary 8-OHdG can be easily detected by the FRET biosensor. The concentrations of 8-OHdG in healthy human urine was in the range of 10–19 nM, which was measured by HPLC [47]. The 8-OHdG concentrations in urine collected from sample 1, sample 2, and sample 3 were in accordance with the normal range. The spike recovery of three samples was 100.2%, 102.5%, and 116.9%, respectively. The method established in the present study is suitable for determining 8-OHdG concentrations in human urine. The concentrations of 8-OHdG in human urine vary with different individuals and the physiological conditions. Moreover, 8-OHdG concentration is relatively low under normal physiological conditions. When there is an imbalance between endogenous oxidants and antioxidants, 8-OHdG concentration is high in human urine [1].

The FRET biosensors based on nanoporous alumina membranes with CDs and Au@ZIF-8 nanoparticles, respectively, as fluorescence donors and signal quenchers are good candidates for 8-OHdG detection with high sensitivity, low LOD, easy operation, and low cost. The FRET biosensors are suitable for urinary 8-OHdG detection and the detection results are in accordance with the HPLC results reported [48]. 

## 4. Conclusions

A sensitive FRET biosensor based on CDs-modified nanoporous alumina membrane was developed with CDs and Au@ZIF-8 nanoparticles as fluorescence donors and signal quenchers, respectively, for detecting 8-OHdG in urine samples. Briefly, 8-OHdG antibody was modified on CDs and Au@ZIF-8 nanoparticles. The capture and detection of 8-OHdG brought Au@ZIF-8 nanoparticles closely to CDs on nanoporous alumina membrane substrate to form the sandwich structure. The fluorescence of CDs can be quenched effectively by Au@ZIF-8 nanoparticles leading to fluorescence intensity decrease. The FRET biosensor can detect 8-OHdG in the concentration range of 0.35 nM to 1.75 μM. The LOD was 0.31 nM. The low LOD made the FRET biosensor applicable for detecting excreted 8-OHdG in urine of healthy person and also patients suffering from various diseases. The experiments on 8-OHdG with various kinds of interference showed good specificity of this FRET biosensor. The developed nanoporous alumina membranes-based FRET biosensors with Au@ZIF-8 nanoparticles as signal quenchers exhibited excellent selectivity, sensitivity, and low LOD. It can be successfully applied for the optical measurement of biomarkers of genotoxicity and some diseases.

## Figures and Tables

**Table 1:** A comparison of 8-hydroxy-2′-deoxyguanosine (8-OHdG) detection by the fluorescence resonance energy transfer (FRET) biosensor with various reported studies.

Method Applied	Materials Used	Detection Range	LOD	References
QCM	Gold surface/MAAP-Fe(III)	0.1–1 mM	12.5 nM	[43]
QCM	MAH-Pt(II)	0.1–1 μM	8.3 nM	[44]
Electrochemical sensor	ZnO@rGO/GCE	5.0–5000.0 nM	1.25 nM	[45]
Voltammetric sensor	MWCNTs/ErGO	3–75 μM	35 nM	[46]
Fluorescence aptasensor	MNPs	3.96–211 nM	1.19 nM	[14]
FRET	Nanoporous alumina membrane/CDs/Au@ZIF-8	0.1–1750 nM	0.31 nM	This work

## Supplementary Materials

The following are available online at https://www.mdpi.com/2079-4991/10/10/2044/s1, Figure S1: XRD pattern of Au@ZIF-8 nanoparticles; Figure S2: FTIR spectrum of Au@ZIF-8 nanoparticles; Figure S3: Energy dispersive X-ray spectroscopy (EDX) result for the carbon dots (CDs)-modified nanoporous alumina membranes with 8-OHdG capture and Au@ZIF-8 nanoparticles attachment; Figure S4: The excitation spectrum and emission spectrum of CDs. Inset: photoluminescence of CDs in solution; Figure S5: Photoluminescence spectra of CDs with pH 2 increased to pH 10. Inset: photoluminescence of CDs with pH 7 (right) and pH 8 (left); Figure S6. The variations of NaCl, KCl, CaCl_2_, MgCl_2_, thymine, cytosine, guanine, and adenine interference.
