# Supplementary material for: A Sensitive FRET Biosensor Based on Carbon Dots-Modified Nanoporous Membrane for 8-hydroxy-2′-Deoxyguanosine (8-OHdG) Detection with Au@ZIF-8 Nanoparticles as Signal Quenchers
Source: Nanomaterials (Basel). 2020 Oct 16;10(10):2044. doi: 10.3390/nano10102044 (PMC7602734; doi:10.3390/nano10102044)
Supplement: Supplementary file 1 [file nanomaterials-10-02044-s001.pdf]

A sensitive FRET biosensor based on carbon dots modified nanoporous membrane for 8-hydroxy-2'-deoxyguanosine (8-OHdG) detection with Au@ZIF-8 nanoparticles as signal quenchers

Weiwei Ye [1,2,*], Yu Zhang [3], Wei Hu [3], Liwen Wang [3], Yu Zhang [4], Kejing Ying [5] and Ping Wang [6]

1 Key Laboratory of E&M, MOE, Zhejiang University of Technology, Hangzhou 310023, China;
2 College of Mechanical Engineering, Zhejiang University of Technology, Hangzhou 310023, China;
3 Department of Food Science and Technology, Zhejiang University of Technology, Hangzhou 310014, China; zhangyuzjut@163.com (Y.Z.); weihu1995@163.com (W.H.); Wlw4712@163.com (L.W.);
4 Mechanical and Automotive Engineering, School of Engineering, RMIT University, Melbourne, Victoria 3004, Australia; fiona.zhang@rmit.edu.au
5 Department of Respiratory Medicine, Sir Run Run Shaw Hospital, Zhejiang University, Hangzhou 310016, China; warsky.com@126.com
6 Biosensor National Special Laboratory, Key Laboratory for Biomedical Engineering of Education Ministry, Department of Biomedical Engineering, Zhejiang University, Hangzhou, 310027, China; cnpwang@zju.edu.cn
* Correspondence: yeweiwei@zjut.edu.com.

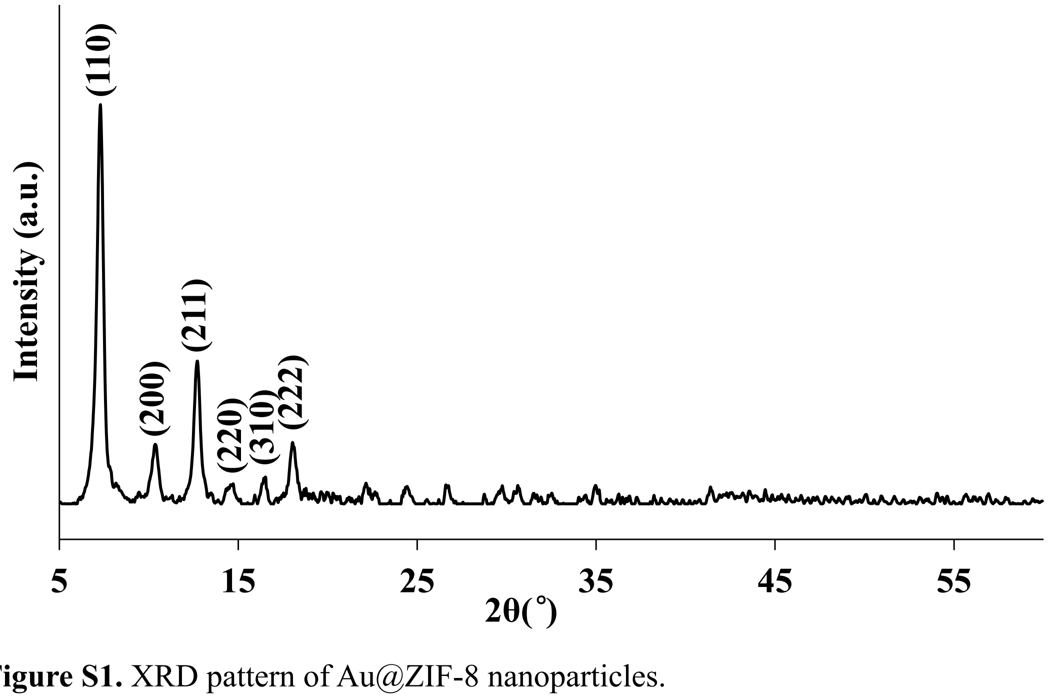

Figure S1. XRD pattern of Au@ZIF-8 nanoparticles.

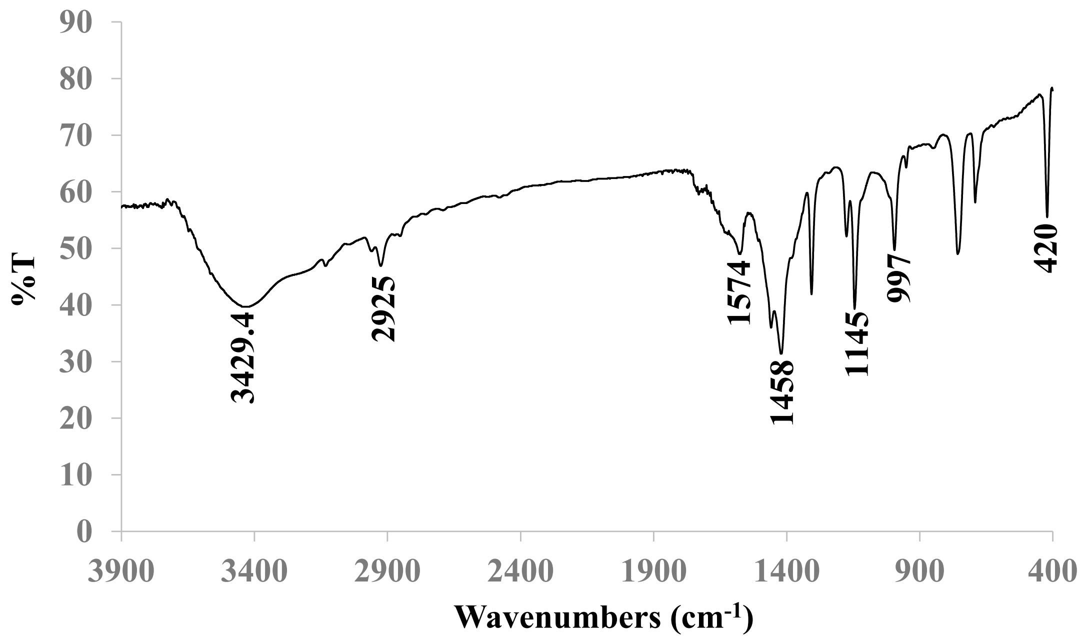

Figure S2. FTIR spectrum of Au@ZIF-8 nanoparticles.

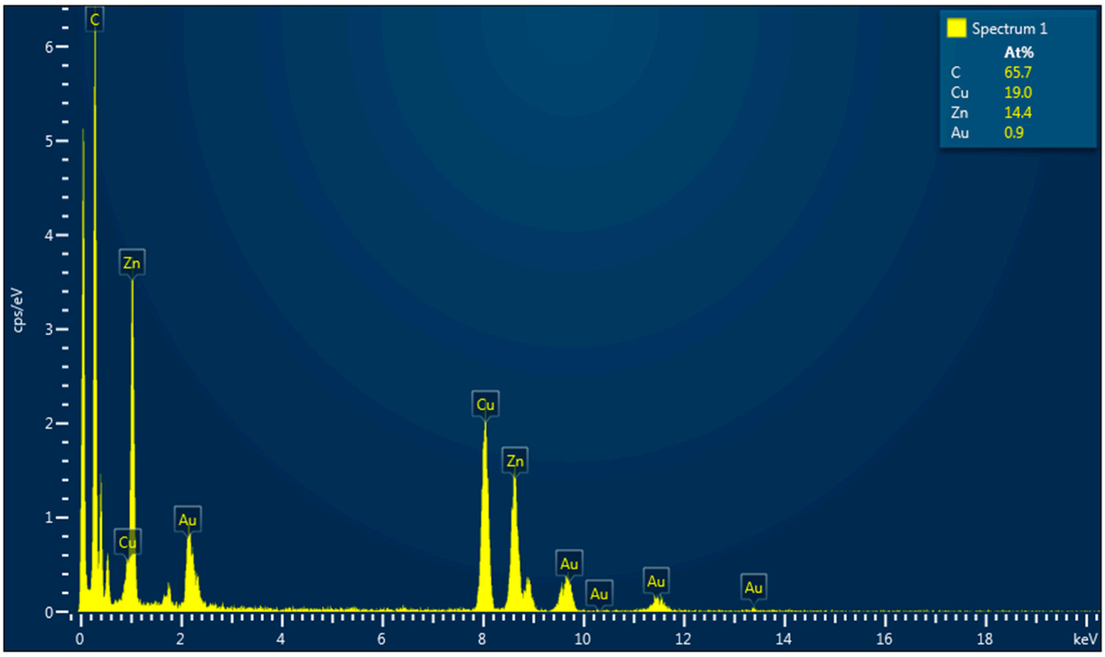

Figure S3. Energy dispersive X-ray spectroscopy (EDX) result for the CDs modified nanoporous alumina membranes with 8-OHdG capture and Au@ZIF-8 nanoparticles attachment.

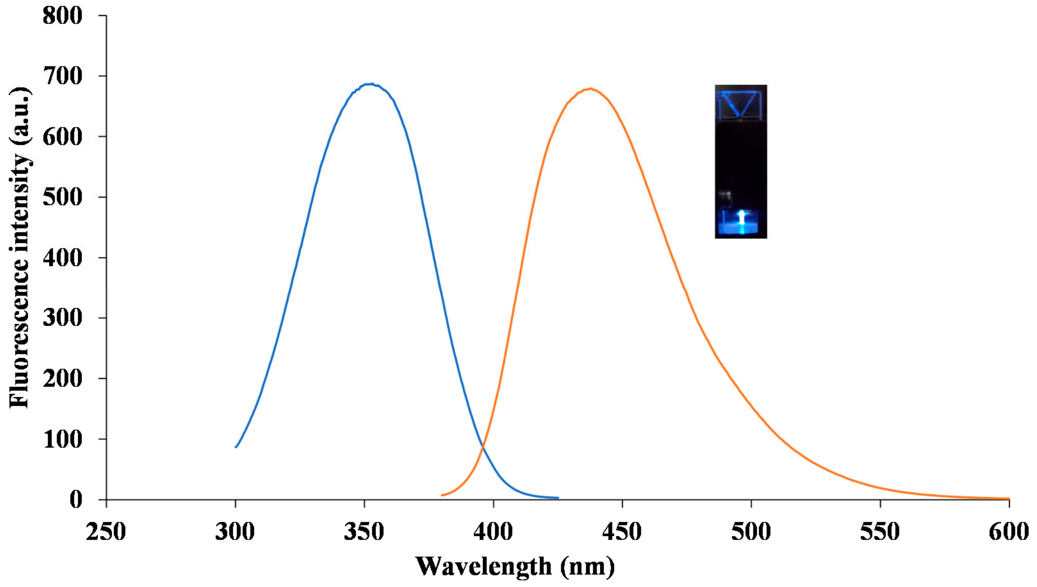

Figure S4. The excitation spectrum and emission spectrum of CDs. Inset: photoluminescence of CDs in solution.

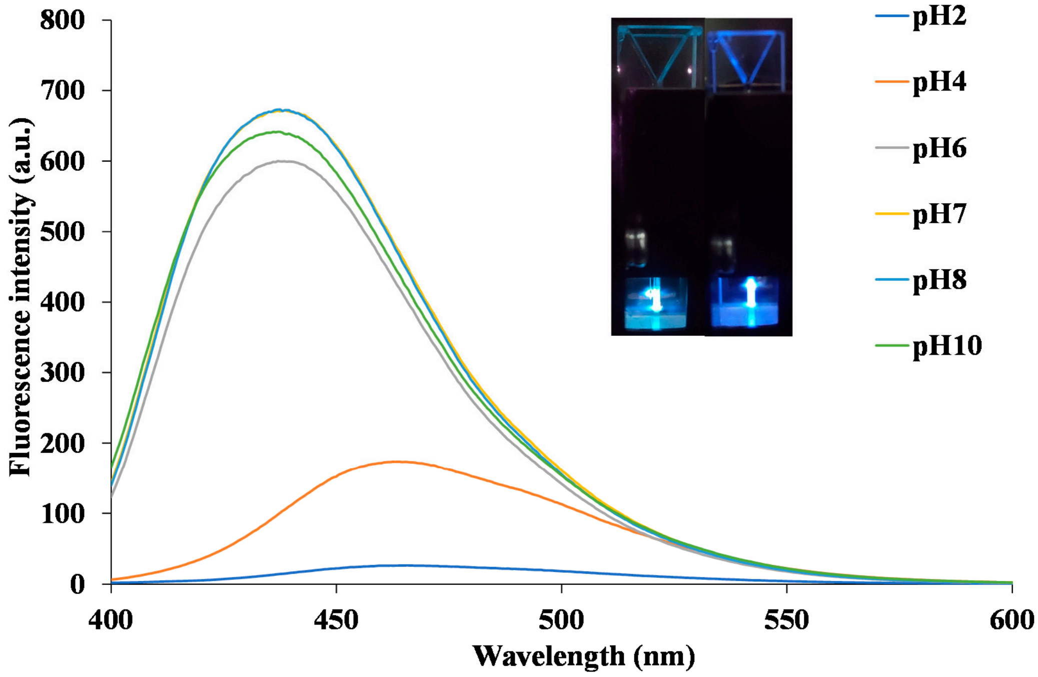

Figure S5. Photoluminescence spectra of CDs with pH 2 increased to pH 10. Inset: photoluminescence of CDs with pH 7 (right) and pH 8 (left).

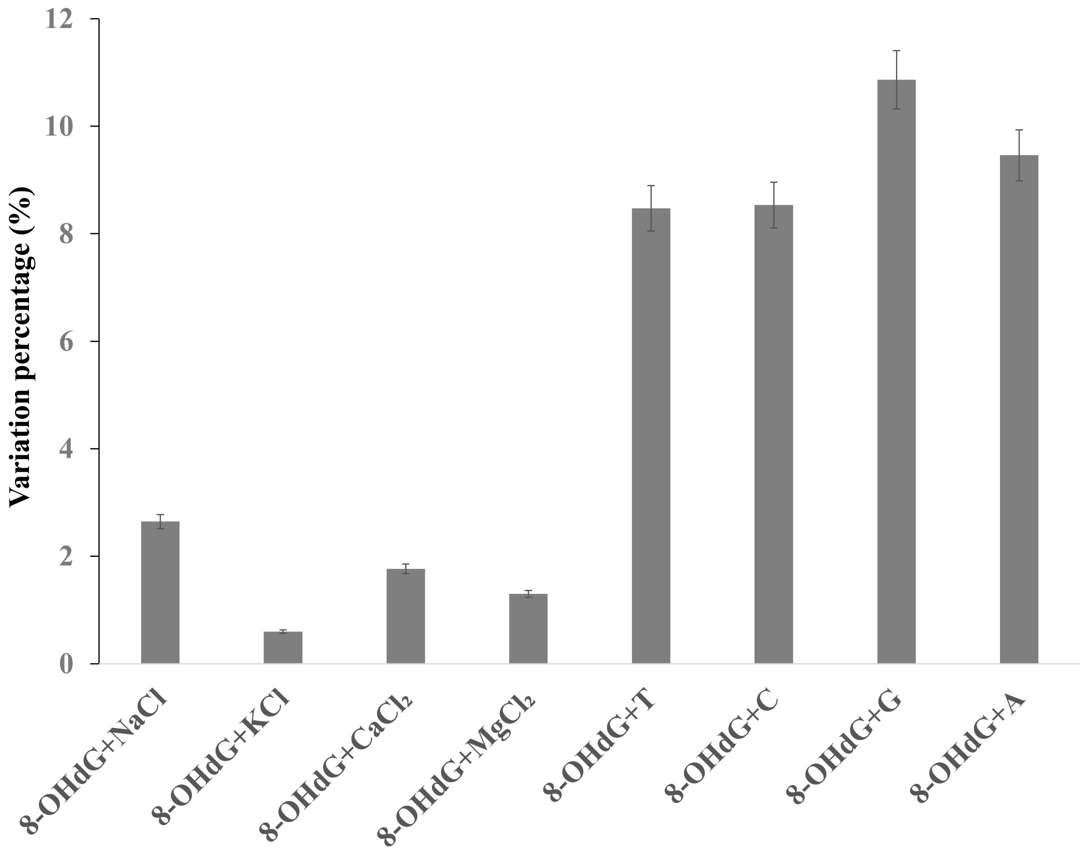

Figure S6. The variations of NaCl, KCl, $CaCl_2$, $MgCl_2$, thymine, cytosine, guanine, and adenine interference.
